# Electrochemical Synthesis of Cu_3_(HHTP)_2_ Metal–Organic Frameworks from Cu Nanoparticles for Chemiresistive Gas Sensing

**DOI:** 10.1021/acsanm.5c02304

**Published:** 2025-07-18

**Authors:** Abigail M. Lister, Ben I. Armitage, Yu Wang, Runze Chen, Weishuo Li, Martin R. Castell

**Affiliations:** Department of Materials, 6396 University of Oxford, Parks Road, Oxford OX1 3PH, United Kingdom

**Keywords:** electrochemical synthesis, metal−organic frameworks, sensors, chemiresistors, 2D materials

## Abstract

The porosity of electrically conductive metal–organic frameworks (MOFs) make them attractive materials for use as the functional sensing element in a variety of electronic devices. Here, we present a route to reliably synthesize conductive MOFs uniformly and in situ through electrochemical growth of Cu_3_(HHTP)_2_ from Cu nanoparticle precursors. The nanoparticles are generated using a magnetron sputtering source and are deposited on glass substrates patterned with interdigitated electrodes. Subsequent solution-based electrochemical growth results in a uniform distribution of the MOF on the substrates as determined through Raman spectroscopy, XPS, SEM, and PXRD techniques. As a proof of concept, the MOF-decorated electrodes are then investigated as chemiresistive sensors for NO_2_ and NH_3_ gases. Sensing of NH_3_ in dry N_2_ carrier gas is achieved with a sub-ppm limit of detection.

## Graphical abstract

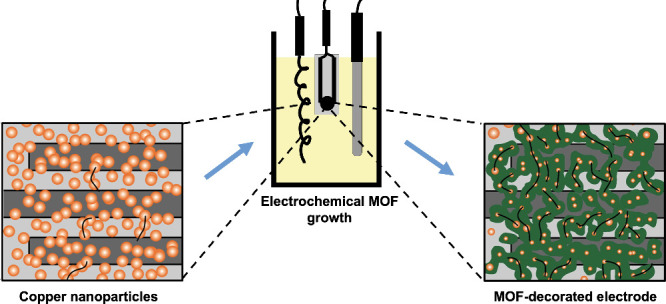

## 1 Introduction

Electrically conductive metal–organic frameworks (MOFs) are conductive porous materials with high specific surface areas and regular pore dimensions.^1–3^ Conductive MOFs are made from metal nodes and organic linkers that join to form 2D layers and then stack to create 3D porous crystals.^4^ Within the layers, the metal centers are present in square planar geometries which enables overlap between the metal d-orbitals and the linker π-systems, resulting in the formation of a continuous delocalized system.^5^ The metal and organic linker units can arrange themselves to form a hexagonal structure such as that depicted for Cu_3_(HHTP)_2_ in Figure 1. A variety of conductive MOFs have been created using diverse metal nodes and ligands.^6^ Because of their advantageous properties, these materials can be applied in numerous fields including energy storage,^7,8^ catalysis,^9,10^ and gas sensing.^11,12^ Of particular note are studies in which the potential use of conductive MOFs in field-effect transistors^13^ and supercapacitors^14^ are reported. However, challenges remain due to the limitations of the existing methods of preparing conductive MOFs for use in electronic devices. Generally, microcrystalline powders of these materials are obtained through the solvothermal method. The prepared powders are then dispersed in solvents and form the active layers through ex-situ coating methods such as drop-casting,^15,16^ spray-coating^17^ or mechanical abrasion.^18^ However, these methods generally result in an uneven distribution of MOF particles on the substrates and a broad range of particle sizes, leading to a significant variability in film resistances. Moreover, the large MOF particle sizes result in poor contact at grain-to-grain and/or grain-to-electrode interfaces. To address these issues, various in situ preparation methods have been developed. These include liquid-phase layer-by-layer epitaxial self-assembly,^19^ vapor-assisted conversion,^20^ and spin-coating interfacial self-assembly.^21^

**Figure 1:**
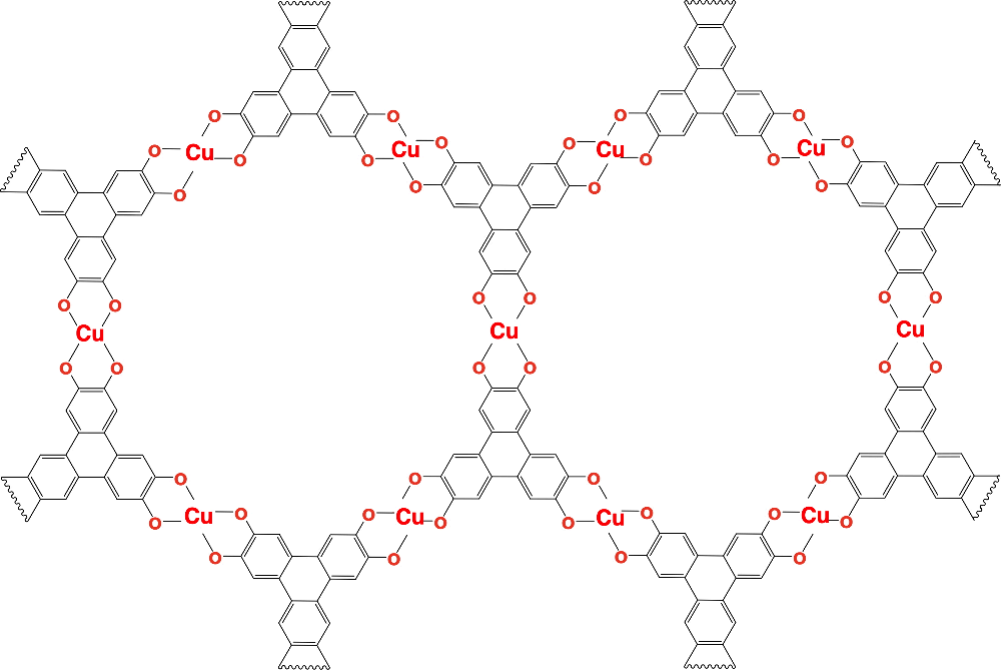
Schematic representation of a single 2D layer of the Cu_3_(HHTP)_2_ MOF. These layers stack to form 3D structures with pore channels.

While the in situ methods described above result in uniformly distributed MOF on the substrate, they are prone to the issue whereby the measured resistance across the electrodes may be dominated by the contact resistance between the particles. Methods to electrochemically grow the MOF have been explored as routes to reduce the effects of contact resistance. Ameloot et al. reported the first electrochemical synthesis of a MOF in 2009 when they synthesized Cu_3_(BTC)_2_ on Cu anodes.^22^ Electrochemical synthesis of Cu_3_(HHTP)_2_ was reported in 2020 when the Bradshaw group described a method to grow MOF adhered to Cu anodes by applying a positive potential in a solution of ligand and electrolyte.^23^ While growing MOF directly on conductive electrodes is useful for functionalized electrode applications,^24–26^ generating the MOF adhered to a conductive substrate such as a Cu anode means that the system cannot be directly used as the active layer in the majority of electronic applications. For example, if this MOF-on-Cu substrate were to be used as a chemiresistive sensor then the resistance would be almost entirely determined by the low electrical resistance of the Cu rather than the resistance of the MOF. This means that even if the resistance of the MOF changes as a function of gas analyte adsorption, the overall resistance of the MOF-on-Cu system would not change by a measurable amount because of the low resistance of the Cu substrate. A route to remove the MOF from the Cu anode was explored by Bradshaw et al., in which poly­(methyl methacrylate) (PMMA) was used to transfer the electrochemically synthesized MOFs to other substrates,^23^ but this procedure is complicated and there is a risk of layer breakage. It is therefore desirable to explore alternative electrochemical methods for MOF preparation that generate the MOF in situ on the desired substrates.

Among many diverse applications, conductive MOFs have shown great potential as sensing layers, because of their porous structures and ability to operate at room temperature. Efforts have been devoted to developing gas sensors based on conductive MOFs,^18,27,28^ and a simple chemiresistor configuration is generally used. These sensors are competitive compared with those based on conjugated polymers and semiconducting metal oxides.^14,29^ MOFs can be systematically designed with particular analytes in mind.^30^ The different MOFs can then be combined into a sensing array to produce a gas sensor with enhanced selectivity, as pioneered by Campbell et al.^18^ In their paper, microcrystalline MOF powders are drop-cast from solution onto interdigitated electrodes (IDEs) in order to make sensors. They are therefore likely to suffer from the problems of high interparticle contact resistance, an uneven MOF distribution leading to a large range of resistances for sensors made using the same method, and a lack of control of MOF placement on the substrate. To make better use of the unique advantages of conductive MOFs in the field of chemiresistive sensors, a straightforward method that produces uniform distributions of the MOF in situ on any desired substrate is required.

Here, we present a route to electrochemically synthesize conductive MOFs from metal nanoparticles and then explore their potential in chemiresistive sensing. Cu_3_(HHTP)_2_, one of the most representative conductive MOFs, is prepared using this method. Cu nanoparticles generated by a magnetron sputtering source are deposited on glass substrates with Pt IDEs, and Cu_3_(HHTP)_2_ is then electrochemically grown on the IDE substrates using chronoamperometry in a solution containing 2,3,6,7,10,11-hexahydroxytriphenylene (HHTP) and a supporting electrolyte. Unlike in previously reported electrochemical MOF synthesis methods, the method outlined here requires no assistance from oxygen bubbling and no removal of the MOF from the substrate in order for it to be used in electronic devices like sensors. An additional advantage of this technique is that growing the MOF from nanoparticles enables control over the regions where the MOF forms through patterning at the nanoparticle deposition stage. Uniform distribution of the MOF is ensured and the coverage of MOF can be controlled by changing the electrochemical synthesis time.

The structure of the prepared Cu_3_(HHTP)_2_ is confirmed through different characterization techniques, including X-ray photoelectron spectroscopy (XPS), Raman spectroscopy and powder X-ray diffraction (PXRD). The morphology of the electrochemically grown MOF is investigated via scanning electron microscopy (SEM). As our method generates a conductive MOF in situ on IDE substrates, we subsequently explore the potential of the generated Cu_3_(HHTP)_2_ for use in gas sensing by assessing its ability to chemiresistively respond to NH_3_ and NO_2_ gases.

## 2 Experimental Details

### 2.1 Nanoparticle Deposition

Cu nanoparticles were deposited onto Pt IDEs with 5 μm gap sizes on glass (Micrux Technologies, Spain). These are comprised of interlaced Pt fingers separated by insulating glass gaps, as illustrated schematically in Figure 2a. Before deposition of any conductive material that bridges the insulating gaps the contact pads have no electrical conductivity between them. An NL50 system manufactured by Nikalyte, Ltd., was used to deposit the Cu nanoparticles onto the IDEs. This instrument is a magnetron sputtering source which uses magnets to trap electrons in the vicinity of the metal target to increase the plasma density and so enable high discharge currents to be accessed even at low pressures and voltages.^31^ 7000 ng cm^–2^ of Cu nanoparticles with diameters between 10 and 50 nm were deposited. If the same amount was deposited as a dense continuous film instead of discrete nanoparticles, this would equate to a thickness of around 8 nm. Further control over the nanoparticle size and uniformity might prove to be advantageous in future studies.

**Figure 2:**
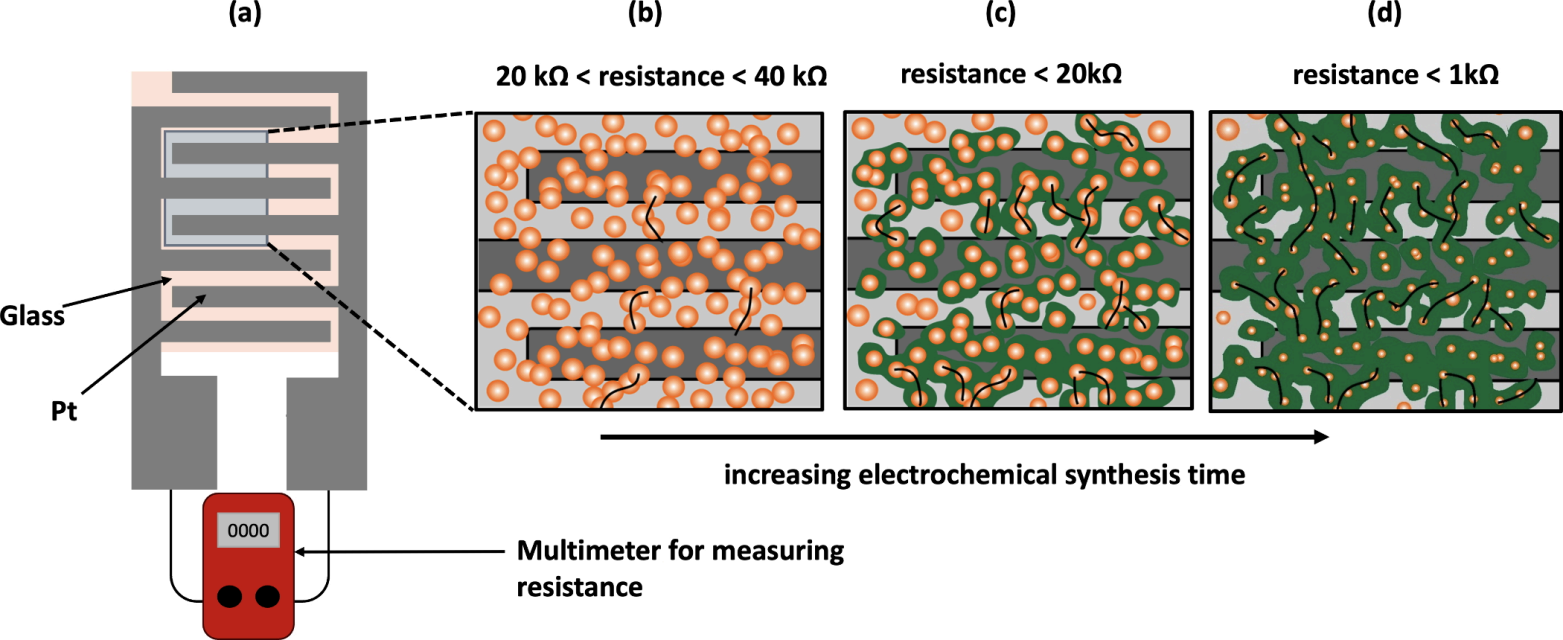
(a) Schematic representation of Pt (gray) on glass (pink) interdigitated electrodes with Cu nanoparticles deposited. (b) Portion of an IDE on which Cu nanoparticles (orange) have been deposited so that limited conductive pathways (black lines) exist between the electrodes. (c) MOF (green) has grown outward from the Cu nanoparticles resulting in more conductive pathways bridging the electrodes. (d) Further electrochemical synthesis showing more MOF and more conductive pathways bridging the insulating gaps resulting in a reduced electrical resistance across the IDE.

The resulting nanoparticle-decorated IDEs had resistances between 100 and 300 kΩ. Each IDE was annealed for 2 h on a hot plate at 200 °C to reduce the resistance to between 20 and 40 kΩ. The aim of this was to improve adhesion of the nanoparticles to the glass of the IDE and improve conductivity by initiating necking between the Cu particles, thereby increasing the proportion of nanoparticles to which the electrochemical potential for MOF formation is applied.

### 2.2 Electrochemical Growth

A 2.6 mM solution of 2,3,6,7,10,11-hexahydroxytriphenylene (HHTP) ligand (Fluorochem, Ltd., U.K.) and 0.021 M solution of tributyl methylammonium methyl sulfate (TBMAMS) electrolyte (Santa Cruz Biotechnology, USA) was made up in a 4:1 by volume ethanol and deionized water mixture. The solution was degassed by bubbling N_2_ gas through the solution for 10 min before the electrochemical potential was applied. A standard 3-electrode setup with a Pt counter electrode (BASi MW-1033 Coiled Platinum Counter Electrode, Alvatek, Ltd., U.K.) and an Ag/AgCl reference electrode (IJ Cambria Scientific, Ltd., U.K.) with a potential of 0.195 V vs the RHE was used. The two sides of the IDE were connected together to form the working electrode and a potential of +0.435 V was applied to cause MOF formation. This was done using a PGSTAT204 Autolab potentiostat (Eco Chemie, The Netherlands) controlled by a PC with NOVA 2.1 software. The potential was applied for times between 1 min and 4 h resulting in samples with different amounts of MOF growth on them.

### 2.3 Characterization Techniques

X-ray photoelectron spectroscopy (XPS), Raman spectroscopy, powder X-ray diffraction (PXRD), and scanning electron microscopy (SEM) were used for sample characterization.

For XPS, a K-alpha instrument from Thermo Scientific was used, and experiments were carried out by the Oxford Materials Characterization Service (OMCS, U.K.). Samples were prepared as described above, applying a potential of +0.435 V to the Cu-decorated IDE for 2 h, while submerging it in the electrochemical growth solution. The MOF was characterized in situ on the IDEs without further processing, and an electron neutralizer was used to prevent charge buildup. An identically prepared sample was used for Raman spectroscopy. This was carried out with a System 1000 instrument from Renishaw, U.K. Scans were performed over a range of 500 to 3000 cm^–1^.

SEM images were taken on an analytical Merlin instrument from Zeiss. The MOF samples were imaged in situ on the IDEs on which they were synthesized. They were synthesized as described above with different samples for each of 0 min, 10 min, and 2 h of electrochemical synthesis time. The Pt contacts of the IDEs were connected to the sample holder with conductive tape to prevent charge build up during imaging. An accelerating voltage of 3 kV was used with a probe current of 100 pA and a working distance of 7 mm. Images were taken with an InLens detector.

PXRD could not be performed with the MOF still adhered to the IDE, and insufficient material could be obtained by scraping MOF off the IDEs. The synthesis was therefore scaled up using bulk Cu tape (RS Pro conductive metallic tape) attached to a glass slide as the Cu source in order to obtain enough material to carry out PXRD. A 4 cm × 4 cm glass slide was covered in Cu tape on both sides and used in place of the nanoparticle-decorated IDE and connected to the electrode setup as described above, where the potential of +0.435 V was applied for 2 h. As observed for the Cu nanoparticle-decorated electrodes, the Cu tape turned black during this time. The black material was then scraped off the foil to yield sufficient powder for the PXRD data to be obtained. The PXRD instrument was a Miniflex from Rigaku, Japan with a 1.54 Å Cu Kα X-ray source. The sample was scanned from 2° to 40° at a speed of 5°/min with a step size of 0.02°.

### 2.4 Sensing Experiments

Sensing tests were performed in a custom-built stainless steel sensing chamber at room temperature and pressure. The gas cylinders used were both purchased from BOC Gases, U.K. Ten ppm ammonia (NH_3_) or nitrogen dioxide (NO_2_) diluted in zero grade dry nitrogen (N_2_) were employed as the analyte gases. The gas flow from each cylinder was controlled using mass flow controllers from Alicat Scientific, USA. The relative flow rates of gases from the two cylinders were controlled via a PC with FlowVision software, so that the analyte gas concentration could be changed. The gas flows from the cylinders were mixed at a T-junction before entering the chamber. Inside the chamber, the sensor was connected to a Model U8001A single-output DC power supply (Keysight, U.K.) to apply a potential of 1 V across the sensor, and to a Model B2900A Source Meter (Keysight, U.K.) to measure the current. The current was monitored in real time on a PC with Keysight Benchvue software.

## 3 Results and Discussion

### 3.1 Preparation and Characterization

The MOF growth process is illustrated schematically in Figure 2, where Figure 2a shows the measurement setup. The preparation of Cu_3_(HHTP)_2_ starts with the deposition of Cu nanoparticles onto clean IDEs using a magnetron sputtering discharge source. Just enough nanoparticles are deposited to take the IDEs from having insulating gaps to having a measurable electrical resistance between 20 and 40 kΩ across them. As shown in Figure 2b, the Cu nanoparticles (orange) deposited on the IDE substrates generate some conductive pathways across the insulating gaps, depicted by black lines. The Cu nanoparticle-decorated IDE substrates are then used to grow the Cu_3_(HHTP)_2_ MOF via chronoamperometry. At the applied potential of +0.435 V, determined by cyclic voltammetry (Figure S1), Cu is ionized to Cu^2+^ through electrochemical oxidation, while HHTP is oxidized from its catechol to semiquinone form. Subsequently, Cu_3_(HHTP)_2_ MOF forms through the coordination between the Cu^2+^ ions and the semiquinone HHTP^3–^ ions. As depicted in green in Figure 2c, the resulting MOF grows outward from the Cu nanoparticles that are experiencing a positive potential due to their electrical connection to the electrodes. This is accompanied by a decrease in size of the nanoparticles as the Cu is consumed to form the MOF. The formation of MOF results in an increase in the number of conductive pathways bridging the electrodes. As the electrochemical synthesis continues, more MOF grows, resulting in more conductive pathways bridging the insulating gaps between the Pt IDEs and a lower measured resistance between the contact pads. The Cu nanoparticles shrink further as they are consumed to form the MOF as shown in Figure 2d.

A number of analytical techniques were used to demonstrate the successful synthesis of the Cu_3_(HHTP)_2_ MOF. X-ray photoelectron spectroscopy (XPS) was carried out in situ on the IDEs on which the MOF was synthesized, with no need to remove the MOF from the substrate. The survey scan shown in Figure 3a shows the presence of the expected carbon, oxygen and Cu elements in the MOF and is consistent with survey scans published in the literature for Cu_3_(HHTP)_2_ MOF.^32^ The intense peaks at 532.1 and 285.1 eV can be attributed to photoelectrons emitted from the O 1s and C 1s subshells, respectively. Also visible, but less intense, are peaks at 168.1 and 232.1 eV. These can be assigned to photoelectrons emitted from sulfur, indicating the presence of some residual TBMAMS electrolyte on the electrode surface. The peak at 402.0 eV is a N 1s peak that is also present in the survey scan of the clean IDE. A higher-resolution scan between 925 and 965 eV reveals the finer structure in the Cu 2p region, as shown in Figure 3b. Significant peak splitting for both the 2p_3/2_ and 2p_1/2_ peaks can be observed, indicating that Cu is present in two valence states. The peaks at 932.4 and 952.2 eV can be assigned to the photoelectrons emitted from the 2p_3/2_ and 2p_1/2_ levels in Cu and Cu^+^, while the peaks at 934.5 and 954.2 eV can be assigned to the same subshells in Cu^2+^. The presence of satellite peaks with similar splitting further confirms the presence of Cu ions.

**Figure 3:**
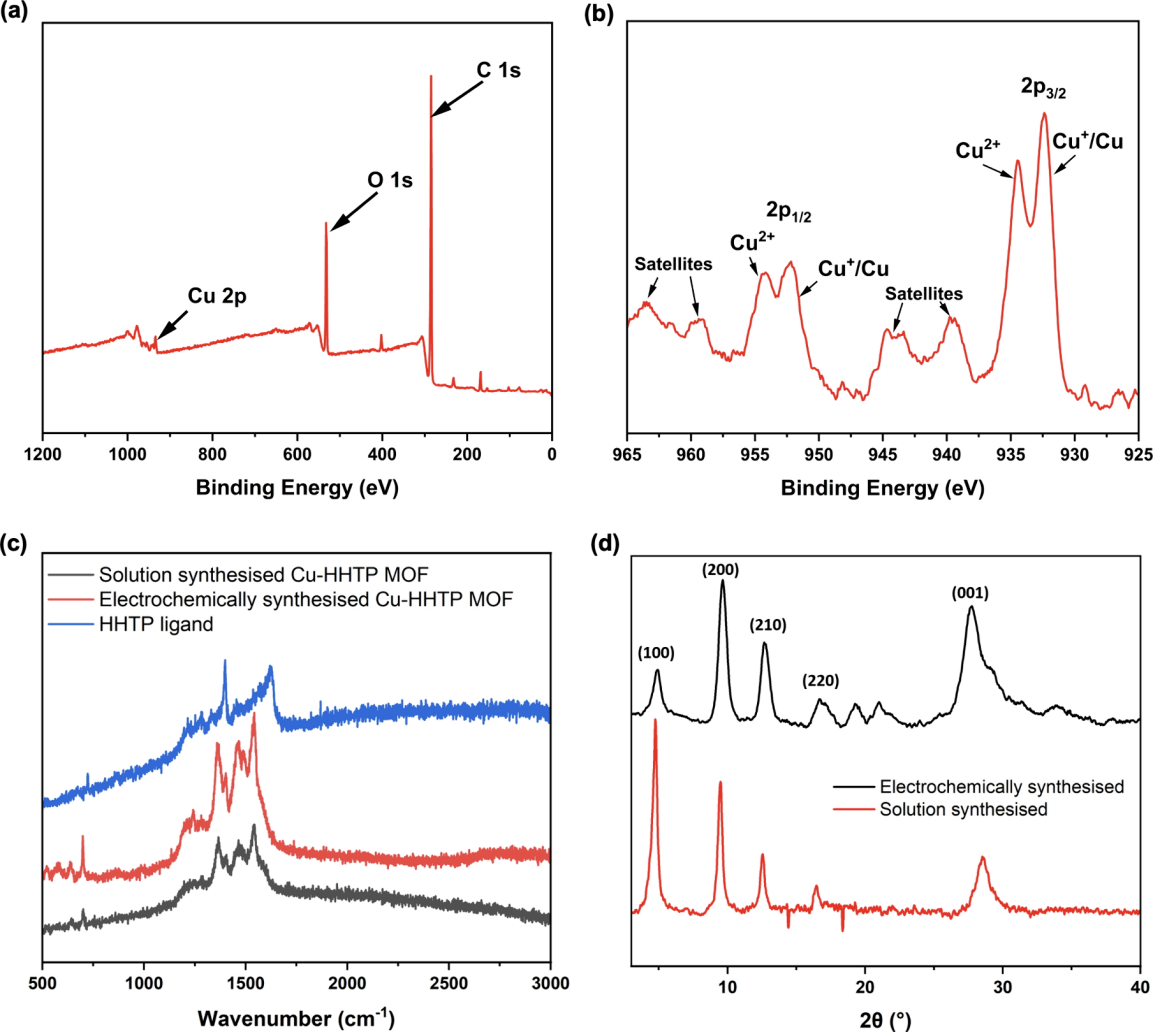
(a) XPS survey scan of Cu_3_(HHTP)_2_ grown on a glass IDE via electrochemical synthesis from Cu nanoparticles. (b) XPS of the Cu 2p region with the Cu^2+^ and Cu^+^/Cu peaks indicated. (c) Raman spectrum of electrochemically synthesized Cu_3_(HHTP)_2_ on glass IDE (red), solution-synthesized Cu_3_(HHTP)_2_ (gray) and HHTP ligand (blue). (d) PXRD spectrum of Cu_3_(HHTP)_2_ synthesized electrochemically from Cu foil (gray) and solution-synthesized Cu_3_(HHTP)_2_ (red).

Raman spectroscopy was carried out with the Cu_3_(HHTP)_2_ still adhered to the glass IDE substrates on which they were synthesized. The resulting spectrum for the electrochemically synthesized MOF can be seen in red in Figure 3c. It has been plotted against the trace for the HHTP ligand starting material (blue) and Cu_3_(HHTP)_2_ MOF synthesized via solution synthesis and drop-cast onto the same IDE substrates (gray). The spectra show the presence of peaks in the MOF scans that are not observed in the spectrum for the HHTP ligand. The spectra have been artificially offset for clarity. On closer inspection of the trace for the electrochemically synthesized sample shown in Figure 3c (red), we observe the most intense peaks at 1363, 1461, and 1542 cm^–1^, with smaller peaks present at 1402 and 1489 cm^–1^, all of which are coincident with the peaks in the solution synthesized MOF spectrum. Modeling is required to fully assign the peaks in the spectra, but comparison to the spectra of other Cu MOFs with similar linkers makes it reasonable to assign the peak at 1542 cm^–1^ to the asymmetric vibration of COO^–^ groups and the peak at 1461 cm^–1^ to the symmetric vibration of the same group.

Sufficient material could not be obtained by scraping MOF off the IDEs for successful PXRD analysis. The synthesis was therefore scaled up as described in section 2.3, allowing the data in Figure 3d (gray) to be obtained. Peaks are observed in the electrochemically synthesized sample at 2θ = 4.95°, 9.64°, 12.67°, 16.68°, and 27.76°. These peaks can be assigned to the (100), (200), (210), (220), and (001) planes, respectively. When indexed to a hexagonal unit cell, the peaks correspond to lattice parameters of *a* = *b* = 21.17 Å, and *c* = 3.21 Å. The small peaks at 19.3° and 21.0° cannot be assigned to any of the reaction mixture components or other potential reaction products. It is therefore assumed that these arise due to impurities in the conductive acrylic adhesive on the Cu tape used for the scaled-up electrochemical synthesis, and we would therefore expect them to be absent on the MOF electrodes prepared from the Cu nanoparticles. In Figure 3d, the PXRD data for the electrochemically synthesized MOF (gray) is plotted alongside the data for the same MOF synthesized via solution synthesis (red). From this we can see that the positions of the indexed peaks are consistent with the peaks in the solution synthesized MOF. They are also consistent with the peak positions reported in the literature.^23,33,34^ In summary, the data in Figure 3 demonstrates that our Cu nanoparticle electrochemical synthesis method produces the MOF shown in Figure 1.

### 3.2 Morphology and Resistance Studies of MOF Growth

By applying the synthesis potential to a series of identical Cu-decorated IDEs for different lengths of time, the progress of the MOF growth could be assessed via SEM imaging. Inspection of Figure 4 allows us to compare the IDE surface before any electrochemical synthesis (the panels labeled “0 min”) with IDEs that have undergone 10 min and 2 h of electrochemical growth. The spotlike nanoparticles visible at 0 min are replaced by broader features with the same random but uniform distribution as the initial nanoparticles after 10 min, suggestive of crystallites nucleating from the nanoparticles. After 2 h, the surface features are bigger still, with the shapes less rounded and more shard-like. The same crystallite growth is observed on the Pt of the IDEs and the glass gaps, which is due to the even deposition of the nanoparticles on both of the electrode components.

**Figure 4:**
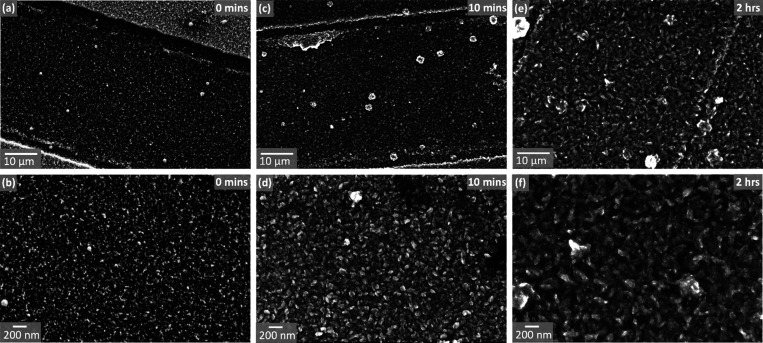
SEM images of Cu nanoparticle-decorated interdigitated electrodes after different electrochemical synthesis times. The left-hand column (panels (a) and (b)) depicts only nanoparticles, whereas Cu_3_(HHTP)_2_ MOF crystals are visible for the samples after 10 min (panels (c) and (d)) and 2 h (panels (e) and (f)) of electrochemical synthesis. Each column depicts the same sample imaged at two different magnifications.

Analysis of the particles depicted in Figures 4b, 4d, and 4f with ImageJ enables average particle sizes to be calculated. The diameters of the as-deposited Cu nanoparticles before MOF formation (Figure 4b) were found to be between 15 and 20 nm. After 10 min of electrochemical synthesis (Figure 4d) the particles have average dimensions of 40–65 nm, while after 2 h of synthesis (Figure 4f), the particles have grown to dimensions of 60–160 nm.

Monitoring the measured resistance across the IDEs provides further evidence for conductive MOF growth between the electrode gaps, with the resistance decreasing with increased electrochemical synthesis time. The plot in Figure 5 was generated through measurement of the resistance between the electrodes of five separate IDEs after different electrochemical synthesis times. The resistance decrease with increased electrochemical growth time is consistent with the MOF growing to bridge gaps between nanoparticles and then further to bridge the insulating gaps in the IDE, as illustrated schematically in Figure 2. The resistance of the sample that underwent 4 h of electrochemical synthesis is however slightly higher than that of the sample synthesized for 2 h. It is expected that there will be a point beyond which the effect of Cu nanoparticles being replaced by the less conductive MOF dominates over the increased bridging between clusters by the MOF. The increase in resistance between 2 and 4 h suggests that this point lies between these two deposition times.

**Figure 5:**
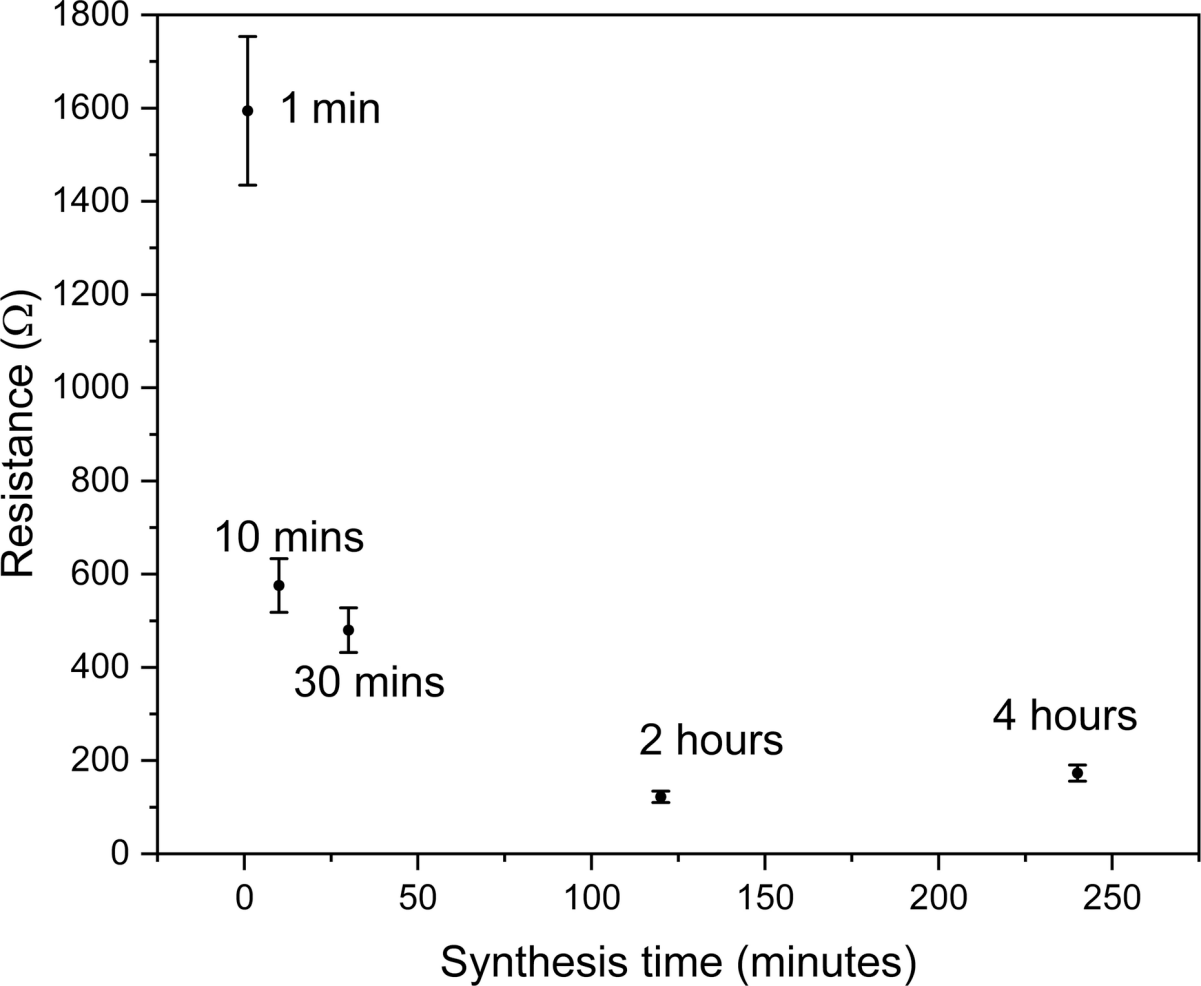
Plot of electrical resistance against MOF electrochemical synthesis time for five different Cu nanoparticle decorated IDEs.

### 3.3 Chemiresistive Gas Sensing

To explore the gas sensing capabilities of the MOF, gas sensing experiments were carried out in which the as-prepared Cu_3_(HHTP)_2_ on IDE samples were used as chemiresistors. Prior to the experiments, the samples were submerged in acetone overnight to draw out any residual solvent from the pores. They were then dried for 10 min on a hot plate at 80 °C before being placed into a custom-built sensing chamber. A potential of 1 V was applied across the IDEs and the current was recorded in real time using a digital multimeter. Ammonia (NH_3_) and nitrogen dioxide (NO_2_) gases were chosen for the experiments to represent reducing and oxidizing analyte gases, respectively. Dry N_2_ was employed as the carrier gas. During the experiments, a constant flow rate of 500 sccm was maintained and the electrical current across the IDEs was monitored.

Figure 6a shows a typical resistance response plot obtained from exposing a chemiresisitive IDE sample that underwent 2 h of electrochemical synthesis to NH_3_ gas. The sample was exposed to concentrations of NH_3_ gas between 5 and 1 ppm for 60 s each. The sensors responded rapidly to analyte gas exposure, with resistance increases starting within 3 s of analyte exposure for all concentrations. The pre-exposure resistance was recovered within 30 min of the analyte exposure ending. Figure 6a shows the prepared Cu_3_(HHTP)_2_ sensor responding to NH_3_ gas with reversible resistance increases. As NH_3_ is a strong electron donor, the resistance increase of the MOF upon exposure to NH_3_ is consistent with the MOF behaving as a *p*-type semiconductor. The response to NO_2_ gas was also probed using the same sensor and the same experimental setup. Irreversible resistance decreases were observed when the same sensor was exposed to the oxidizing gas NO_2_ (Figure S2). The contrasting response characteristics of the NH_3_ and NO_2_ exposures demonstrates that the fabricated sensor can distinguish between the two gases. As the introduction of NO_2_ gas led to an irreversible change in resistance, the sensing response magnitude decays too much with successive exposures for an accurate limit of detection to be calculated. For the NH_3_ responses, however, the sensing response percentages for various concentrations of NH_3_ gas were obtained by calculating the change in resistance on exposure as a percentage of the initial resistance (Δ*R*/*R*_0_ × 100%). A linear relationship was observed between the sensing response and the concentration of NH_3_ gas (Figure 6b). The sensitivity of the sample, defined as the slope of the linear fit in Figure 6b, is 0.154 ± 0.006% ppm^–1^. The limit of detection (LOD) is then defined as three times the root-mean-square (RMS) of the baseline noise divided by the sensitivity. For the sample in Figure 6, this gives a theoretical LOD of 158 ± 6 ppb. The same sensor was exposed to 3 ppm of NH_3_ again after 1 year in storage in ambient conditions. After this time, the resistance of the sample increased from 183 Ω to 1245 Ω and the response to 3 ppm of NH_3_ actually increased from 0.41% to 12% (Figure S3).

**Figure 6:**
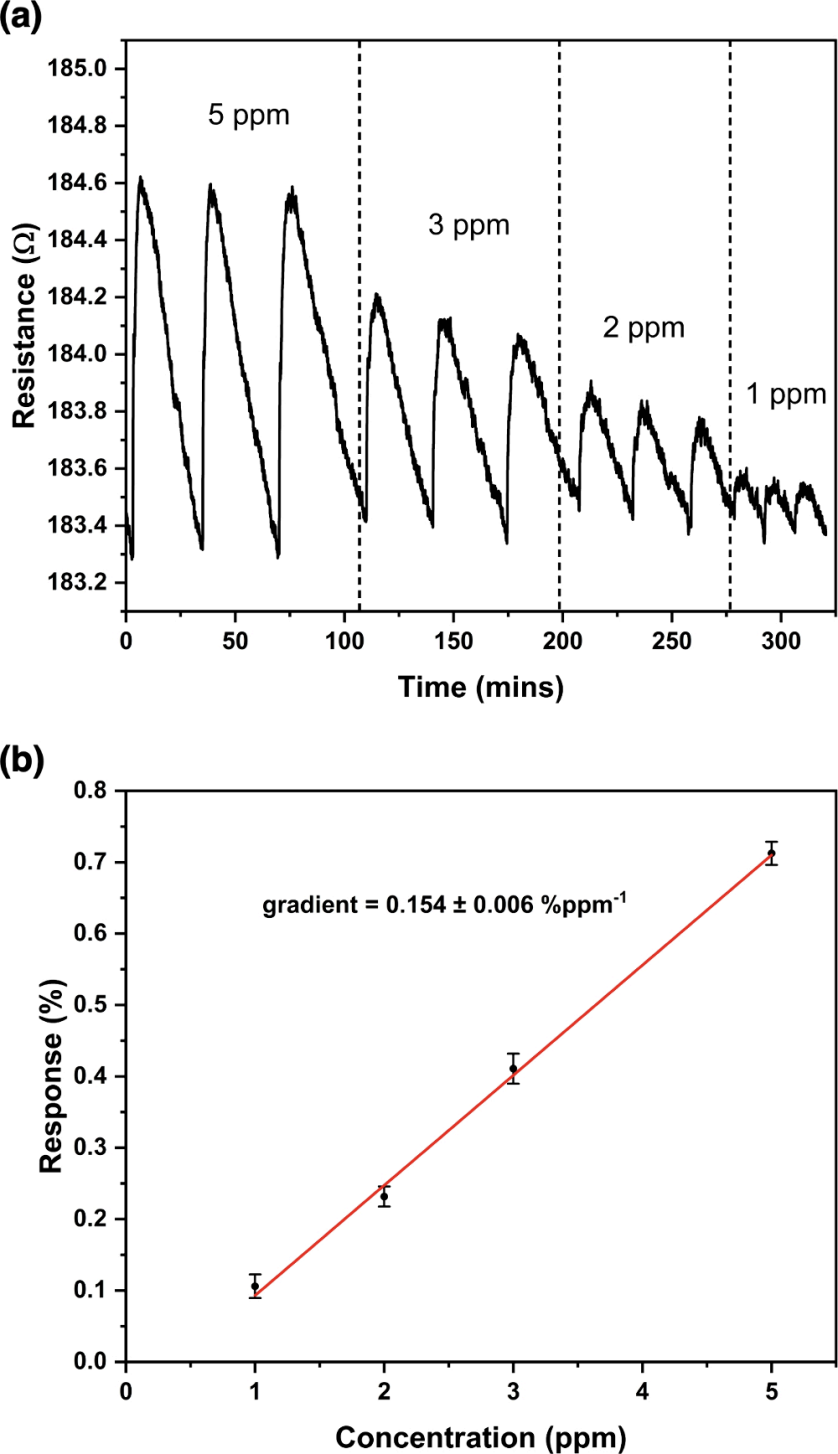
NH_3_ in dry N_2_ carrier gas sensing results obtained for a Cu_3_(HHTP)_2_ MOF chemiresistive sensor at room temperature and ambient pressure. (a) The resistance response of the sensor exposed to NH_3_ gas with concentrations between 5 and 1 ppm for 60 s. (b) Plot of the linear relationship between response (%) and NH_3_ concentration (ppm). The gradient of this plot gives the sensitivity of the sensor.

A chemiresistive Cu_3_(HHTP)_2_ NH_3_ sensor with a calculated limit of detection was fabricated by Yao et al. using a spray layer-by-layer deposition method. They report an LOD of approximately 500 ppb.^19^ Our calculated LOD of 158 ppb is competitive with the LODs quoted for chemiresistive sensors fabricated from conducting polymers and metal-oxide semiconductors.^29^ However, it should be noted that factors such as the gas flow rate, exposure time, and sensing chamber geometry have significant effects on the sensing responses and limits of detection that are determined. Comparisons between sensors made by different research groups should therefore be treated with caution, as experimental conditions are rarely consistent. Experiments performed within our research group using solution-synthesized Cu_3_(HHTP)_2_ drop-cast onto the same IDEs, found the LOD to be 1200 ± 200 ppb under the same sensing conditions (Figure S4). This result shows that the electrochemically synthesized MOF described here is superior to the analogous solution-synthesized MOF sensors in terms of both LOD and linearity of the response.

While the primary focus of this paper is the electrochemical synthesis method of the MOF using metal nanoparticles, the sensing results are a proof-of-principle that the MOF made by our method can be directly employed in chemiresistive sensing. Future work will aim to achieve a systematic understanding of the relationship between synthesis conditions and sensor performance. These future studies will address potential limitations of the unoptimized sensor presented here, such as the recovery time and response repeatability for NH_3_ sensing. The effects of temperature, humidity, and device to device variation on sensor performance will also be investigated.

## 4 Conclusion

A method to electrochemically grow Cu_3_(HHTP)_2_ from prepatterned Cu nanoparticles has been presented. The protocol allows us to prepare the conductive MOF in situ on IDEs. SEM imaging indicates that significant MOF growth occurs within the first 10 min of applying the electrochemical potential, which is corroborated by a decrease in measured electrical resistance across the electrodes. Growing the MOF from nanoparticles enables control over the regions where the MOF forms through patterning at the nanoparticle deposition stage. Uniform distribution of MOF is ensured and the coverage of the MOF can be controlled by changing the electrochemical synthesis time. As the method forms a conductive MOF in situ that is strongly adhered to an insulating substrate, it is anticipated to be beneficial for integration of Cu_3_(HHTP)_2_ MOF into a variety of electronic devices. For example, we demonstrate how fabricating Cu_3_(HHTP)_2_ with the described method can be used to form a chemiresistive sensor able to detect NH_3_ gas with a limit of detection of 158 ± 6 ppb and sensitivity of 0.154% ppm^–1^.

Compared with previously reported methods of electrochemically growing Cu_3_(HHTP)_2_ adhered to Cu anodes,^23^ the method described here provides attractive attributes in terms of uniformity of chemiresistive sensing medium, control of MOF growth, and reduced post-growth processing requirements. While some other electrochemical methods have been reported that also do not require growth of the MOF adhered to Cu anodes, they require strict conditions such as continuous O_2_ bubbling and precisely controlled pHs.^35,36^ Furthermore, the method has potential to be applicable for the preparation of a variety of MOFs on different substrates, as the metal nanoparticle sputtering and electrochemical growth techniques can be broadly applied to many combinations of metals and organic ligands. It is anticipated that our method can be successfully applied to a broad range of substrates, with potential applications in flexible and wearable sensing technologies.

## Supplementary Material



## Notes

Cyclic voltammetry, sensing responses of samples to nitrogen dioxide, sensing responses of Cu_3_HHTP_2_ powder to ammonia (PDF)
