# Supplementary material for: Electrochemical Synthesis of Cu3(HHTP)2 Metal–Organic Frameworks from Cu Nanoparticles for Chemiresistive Gas Sensing
Source: ACS Appl Nano Mater. 2025 Jul 18;8(30):15114–21. doi: 10.1021/acsanm.5c02304 (PMC12322875; doi:10.1021/acsanm.5c02304)
Supplement: Supplementary file 1 [file an5c02304_si_001.pdf]

Supporting Information for

Electrochemical Synthesis of $Cu_3(HHTP)_2$ Metal-Organic Frameworks from Cu Nanoparticles for Chemiresistive Gas Sensing

Abigail M. Lister, Ben I. Armitage, Yu Wang, Runze Chen, Weishuo Li and Martin R. Castell**

Department of Materials, University of Oxford, Parks Road, Oxford OX1 3PH, United Kingdom.

*email: abigail.lister@materials.ox.ac.uk; martin.castell@materials.ox.ac.uk

1. Cyclic Voltammetry

Cyclic voltammetry (CV) was performed to investigate the redox processes involved with formation of the MOF. An IDE with copper nanoparticles deposited on it was submerged in a solution of the ligand and electrolyte and the potential was ramped from -0.80 V to +1.20 V in steps of 0.00244 V at a speed of 0.02 V s^{-1}. The two sides of the IDE were connected for this experiment, and the current flowing between the IDE working electrode and the reference electrode is recorded as the voltage is changed. The resulting current vs potential plot is given in Figure S1.

In the forward direction, peaks occur at 0.148 and 0.766 V. The first of these is assumed to be due to the oxidation of metallic Cu to Cu^+ ions, whilst the second is likely due to the further oxidation of Cu^+ to Cu^{2+}. In the reverse direction, we observe a positive current peak at 0.579 V and a negative peak at 0.140 V. The peak at 0.140 V can be attributed to the reduction of Cu^+ ions back to Cu metal. The peak at 0.579 V that crosses over the forward scan, indicates that electrons are being donated to the electrode despite the fact that the potential is being decreased. We postulate that this peak corresponds to Cu^{2+} ions oxidising the HHTP molecules. On the forward scan Cu^{2+} ions form which can then participate in the step-wise oxidation of the hydroxy-substituted ligand to the semiquinone species. Each Cu^{2+} ion can accept one electron, forming Cu^+, and so oxidise one dihydroxy group to the corresponding semiquinone radical. Since this involves the copper species on the electrode accepting electrons which can then be transferred to the circuit, the peak presents in the positive current direction crossing over the forward traces. As the solution is degassed with N_2 flow prior to the experiment, O_2 dissolved in the solution cannot participate in ligand oxidation. The CV scans suggests that the maximum rate of ligand oxidation occurs at a potential of 0.579 V, with a lower rate at higher potentials in the reverse direction due to the lower stability of the Cu^+ ion in this environment.

The insights from the CV experiments inform the mechanism of MOF formation, resulting in the following sequence of steps:

1. Oxidation of Cu to Cu^{+} and then Cu^{2+} due to the applied potential.
2. Deprotonation of the ligand in solution.
3. Oxidation of the ligand by Cu^{2+} ions on the IDE, forming Cu^{+}.
4. Deprotonation of the ligand and combination with Cu ions to irreversibly form the MOF.

The 0.435 V applied for MOF formation, as marked by the dashed line in Figure S1, is approximately at the point where the Cu^{+}/Cu^{2+} current increase begins. It is therefore likely that this is the minimum potential that can be used to trigger formation of the Cu^{2+} ions, whilst minimising the oxidation rate, which is desirable for formation of larger crystals.

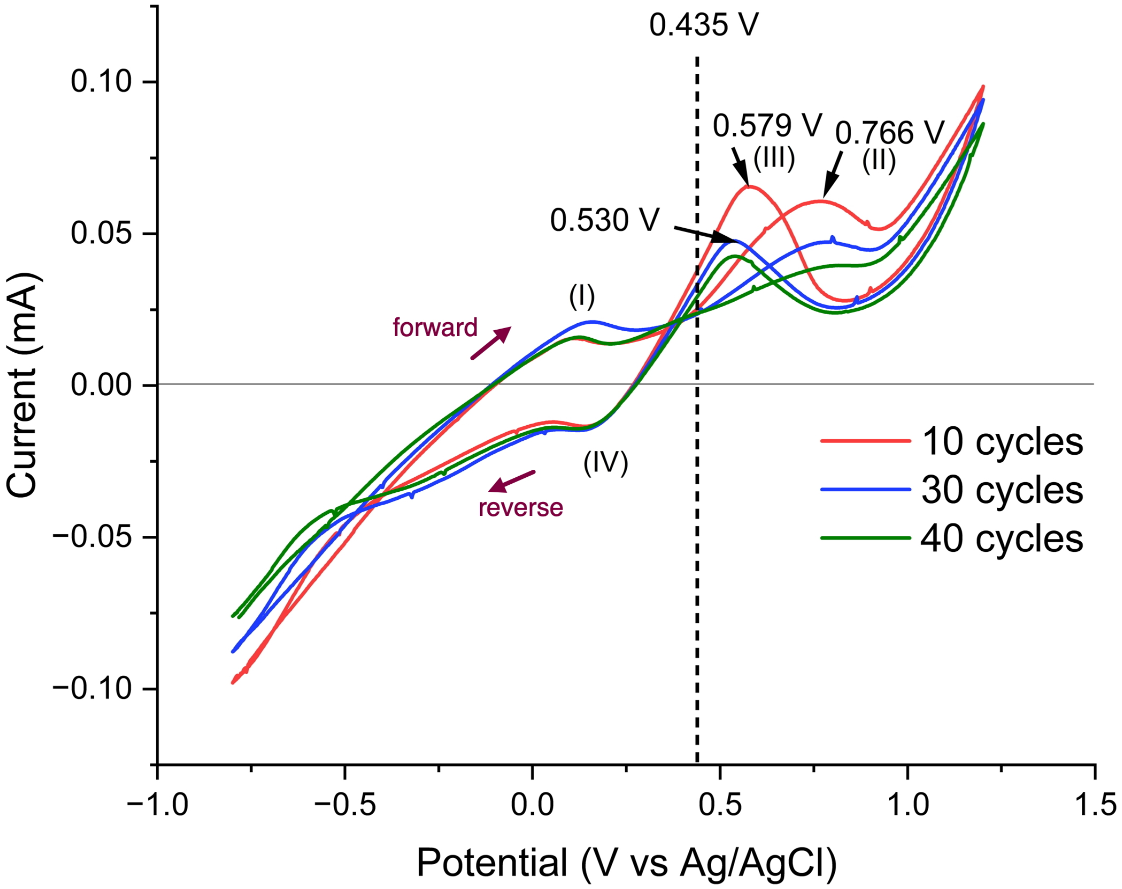

Figure S1. Cyclic voltammogram for a copper nanoparticle-decorated electrode in a solution of HHTP ligand and electrolyte. The potential was ramped from -0.8 V to +1.2 V for 40 cycles. The 10th, 30th, and 40th cycles are shown in red, blue, and green, respectively. Peaks in the forward direction occur at 0.15 V indicated by (I) and 0.77 V indicated by (II). Peaks in the reverse direction appear between 0.58 V and 0.53 V indicated by (III), and 0.14 V indicated by (IV).

2. Sensing Response to NO_2

A $Cu_3(HHTP)_2$ sensor was prepared by subjecting a copper nanoparticle-decorated IDE to 2 hours of electrochemical synthesis, as described in section 3.1 of the main manuscript. The sample was exposed to concentrations of NO_2 gas between 5 and 1 ppm for 60 s per exposure. Figure S2 shows the $Cu_3(HHTP)_2$ sensor responding to NO_2 gas with irreversible resistance decreases. As NO_2 is an oxidizing gas, the observed lowering in resistance of the $Cu_3(HHTP)_2$ MOF is consistent with the MOF behaving as a p-type semiconductor.

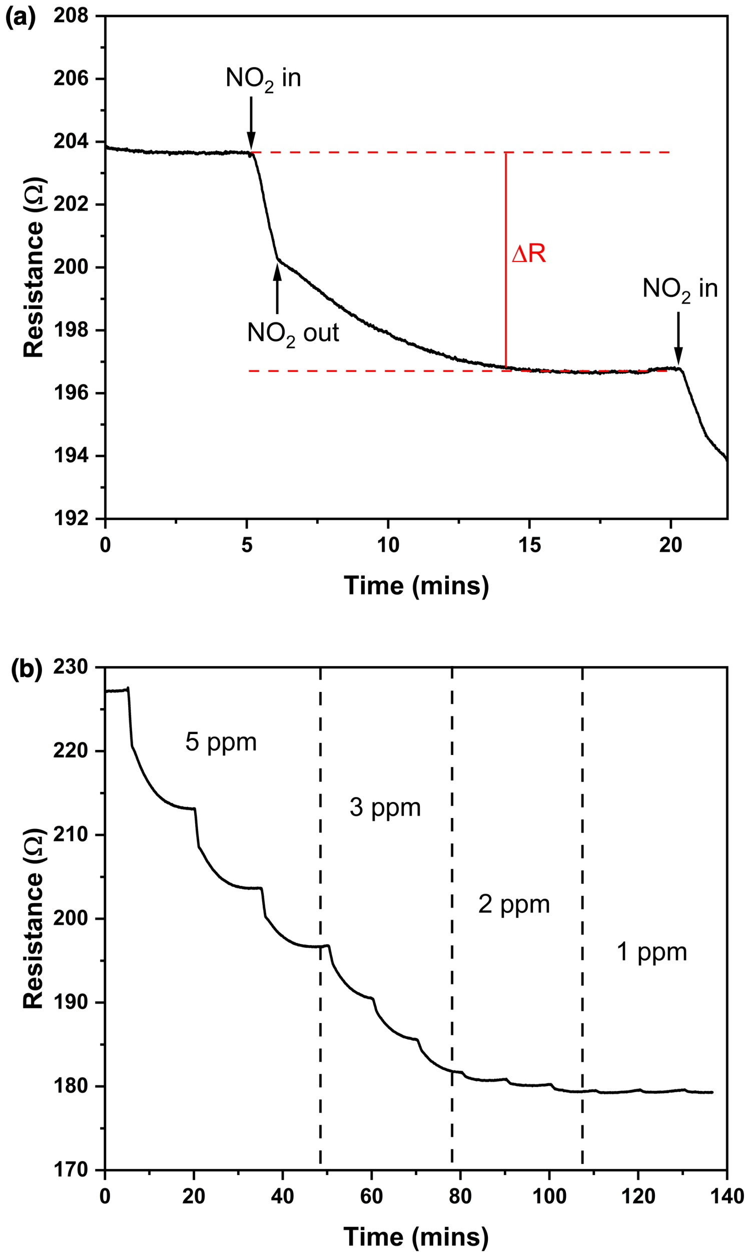

Figure S2. The irreversible resistance response of an electrochemically synthesised $Cu_3(HHTP)_2$ sensor exposed to NO_2 gas. (a) Plot of a single 5 ppm NO_2 exposure indicating the start and end points of the exposure and the resulting lowering of the resistance by ΔR. (b) Multiple exposures with concentrations between 5 and 1 ppm for 60 s per exposure.

3. Long-term stability

The same sample that was used to obtain the data in Figure S2 and Figure 6 in the main manuscript was exposed to 3 ppm of NH_3 gas 12 months after the original experiments were performed. The sample was stored in ambient conditions in the laboratory for one year, during which the resistance increased from 183 Ω to 1245 Ω. The percentage change in resistance on 3ppm NH_3 exposure for this aged sensor had an initial value of 12 % as shown in the first peak in Figure S3. Subsequent exposures to 3 ppm NH_3 show that the response decreases on repeated exposures, reaching a response of 5.6 % on the fourth exposure in Figure S3. Such significant decreases in response between exposures of the same NH_3 concentration were not observed when the sample was first synthesised.

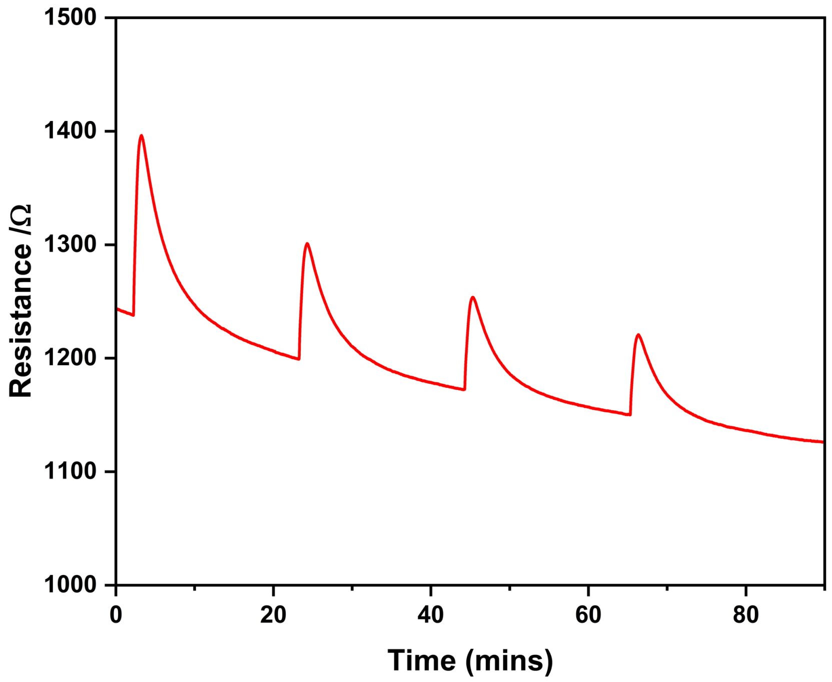

Figure S3. The resistance response of an electrochemically synthesised $Cu_3(HHTP)_2$ sensor that has been stored in ambient conditions for one year since its first use. The sensor is exposed to 3 ppm of NH_3 gas 4 times and shows reversible resistance increases on each occasion. The response magnitude decreases with subsequent exposures.

4. Solution-synthesized MOF NH_3 Sensing

A sensor was prepared by drop-casting 30 × 5 μL drops of 10 mg mL^{-1} $Cu_3(HHTP)_2$ in IPA onto the same type of Pt/glass interdigitated electrodes (IDEs) as were used for the electrochemical synthesis. This resulted in a total of 1.5 mg of MOF being deposited on the IDE. The sensor was submerged in acetone overnight and then dried for 10 minutes on a hot plate at 80 °C. The initial sensor resistance was 4450 Ω. A potential difference of 1 V was applied across the IDEs and the current flow was recorded as the sample was exposed to 60 s exposures of NH_3 gas with concentrations between 1 ppm and 5 ppm. The results of this experiment can be seen in Figure S4a, which shows reversible resistance increases on exposure to NH_3. The magnitude of the resistance changes is much smaller relative to the baseline drift than those observed for the electrochemically-synthesized sensor. The data is presented in Figure S4b after baseline subtraction.

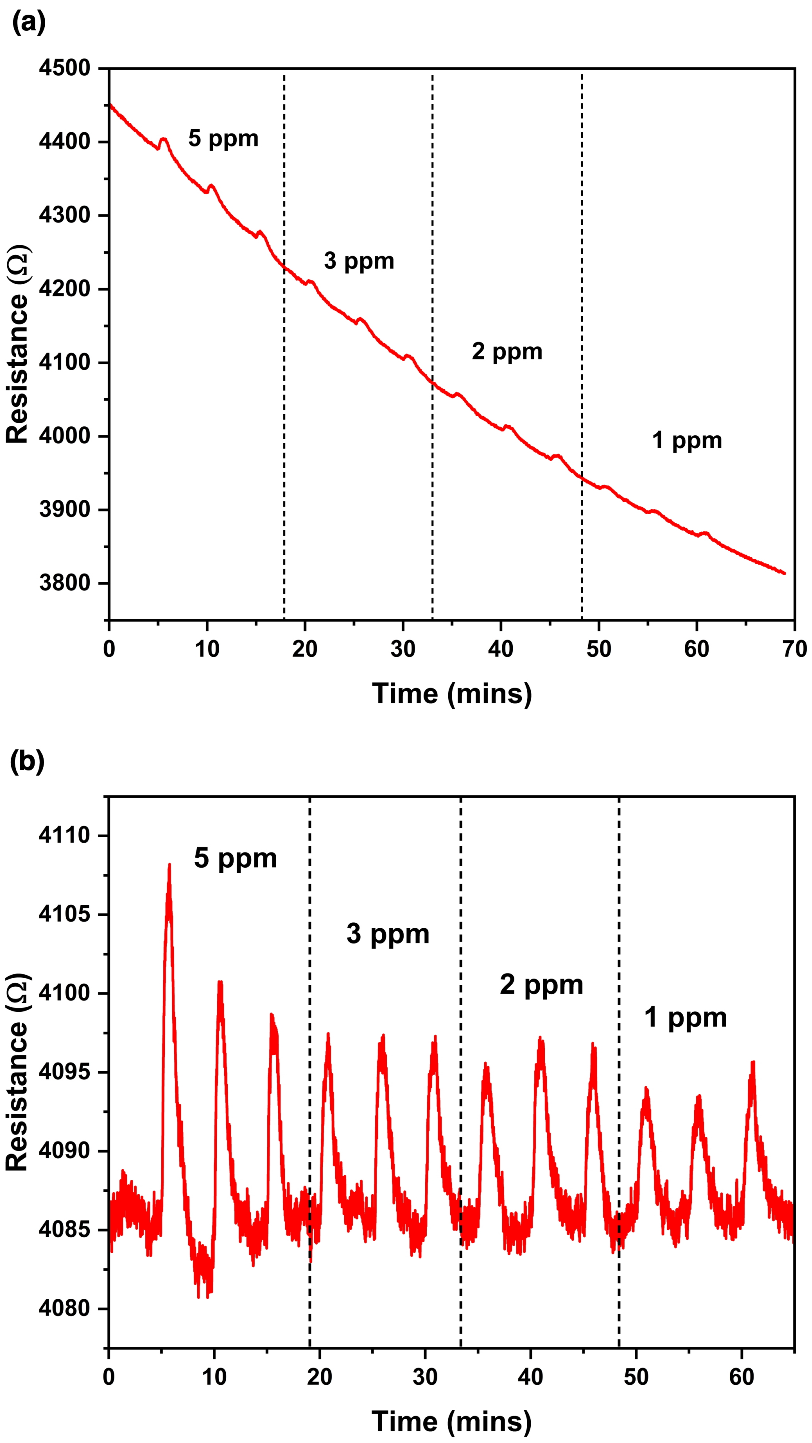

Figure S4. (a) Resistance change of a MOF sensor made from drop-cast solution-synthesized $Cu_3(HHTP)_2$ when it is exposed to NH_3 gas with concentrations between 5 and 1 ppm. (b) Baseline-subtracted resistance change of the sensor exposed to NH_3 gas.
